# It is time to classify biological aging as a disease

**DOI:** 10.3389/fgene.2015.00205

**Published:** 2015-06-18

**Authors:** Sven Bulterijs, Raphaella S. Hull, Victor C. E. Björk, Avi G. Roy

**Affiliations:** ^1^Faculty of Science, Ghent UniversityGhent, Belgium; ^2^Heales vzwBrussels, Belgium; ^3^Biochemistry Department, University of OxfordOxford, UK; ^4^The Biogerontology Research FoundationLondon, UK; ^5^Institutionen för Biologisk Grundutbildning, Uppsala UniversityUppsala, Sweden; ^6^Institute for Translational Medicine, School of Science, University of BuckinghamBuckingham, UK

**Keywords:** aging, nosology, disease, medicine, philosophy of medicine, multisystemic disease, disease complex

## History of disease definitions

What is considered to be normal and what is considered to be diseased is strongly influenced by historical context (Moody, 2001/2002). Matters once considered to be diseases are no longer classified as such. For example, when black slaves ran away from plantations they were labeled to suffer from drapetomania and medical treatment was used to try to “cure” them (Reznek, 1987). Similarly, masturbation was seen as a disease and treated with treatments such as cutting away the clitoris or cauterizing it (Reznek, 1987). Finally, homosexuality was considered a disease as recently as 1974 (Reznek, 1987). In addition to the social and cultural influence on disease definition, new scientific and medical discoveries lead to the revision of what is a disease and what is not (Butler, 2008). For example, fever was once seen as a disease in its own right but the realization that different underlying causes would lead to the appearance of fever changed its status from disease to symptom (Reznek, 1987). Conversely, several currently recognized diseases, such as osteoporosis, isolated systolic hypertension, and senile Alzheimer's disease, were in the past ascribed to normal aging (Izaks and Westendorp, 2003; Gems, 2011). Osteoporosis was only officially recognized as a disease in 1994 by the World Health Organization (WHO, 1994).

## Current definitions of disease

Disease is a complex phenomenon and a current definition must consider both a biological and social explanation. The medical definition of disease is any abnormality of bodily structure or function, other than those arising directly from physical injury; the latter, however, may open the way for disease (Marcovitch, 2009). The disorder has a specific cause and recognizable signs and symptoms, and can affect humans, other animals, and plants (Martin, 2010). The social aspect of disease is significant when trying to divide a line between a healthy and a pathological state. This is a highly context and value driven process and, considering the WHO definition of health as a “state of complete physical, mental, and social well-being and not merely the absence of disease or infirmity,” it is not as simple as classifying disease as the opposite of health (WHO, 1946). “Someone starving to death is not taken to have a disease, but is still not considered healthy” (Reznek, 1987).

Reznek (1987) shows that diseases do not constitute a “natural kind.” If diseases were to share a particular real essence in virtue of which they are all diseases, an unidentified disease could be classified simply by comparing it to an already known pathology and seeing if it fell into the same natural kind as, for example, tuberculosis or cancer. What is a disease and what is not is thus something we invent to create a classification of medically-treatable conditions. In many cases, diseases also seem to lack a nominal sense, as there are no necessary and sufficient conditions that they must have to qualify as diseases, rather than as separate pathological conditions such as injuries, disabilities, and deformities.

Despite it being difficult to define disease as a whole, we can simplify our definitions of disease and our approaches to their treatment by grouping particular pathologies together. For example, cholera, tuberculosis, and pneumonia are all separate diseases in their own right but can be collectively treated as bacterial diseases. The case is similar for HIV, influenza, and measles—all individual impairments to a normal state of health, yet grouped together as viral diseases. Having these classes of disease makes initial strategies for treatment far simpler, which, above all, is the main reason for having a definition(s) of disease in the first place. Could we not take the same approach and add in a further group to treat the aging process as a disease in itself?

## Is aging a disease?

Traditionally, aging has been viewed as a natural process and consequently not a disease (Callahan and Topinkova, 1998; Hayflick, 2007). This division may have, in part, originated as a way of establishing aging as an independent discipline of research (Blumenthal, 2003). Some authors go as far as to create a division between intrinsic aging processes (termed primary aging) and diseases of old age (termed secondary aging) (Hazzard, 2001). For example, photoaging, the accelerated deterioration of skin as a result of UV rays during one's lifetime, is considered by dermatologists as a condition leading to pathology (Rabe et al., 2006). In contrast, chronological skin aging is accepted as the norm. As well as being seen as separate from disease, aging is looked at as a risk factor for developing disease (Hayflick, 2002; Collier et al., 2011; Niccoli and Partridge, 2012). Interestingly, the so-called “accelerated aging diseases” such as Hutchinson-Gilford Progeria Syndrome, Werner syndrome, and Dyskeratosis Congenita are considered diseases. Progeria is considered a disease but yet when the same changes happen to an individual 80 years older they are considered normal and unworthy of medical attention (Caplan, 2005).

The reason commonly cited against classifying aging as a disease is that it constitutes a natural and universal process, while diseases are seen as deviations from the normal state (Caplan, 1992). The distinction between natural and unnatural may depend on the notion of design, purpose, and function (Hausman and Kennedy, 1975; Becker and Becker, 2001). Evolutionary theory of aging teaches us that aging is caused by the decrease in the force of selection against alleles with deleterious effects later in life (Williams, 1957). Aging is thus the consequence of evolutionary neglect, not evolutionary intent (Olshansky et al., 2002). If aging serves no purpose then the notion of aging as a natural process might be mistaken (Caplan, 2005).

Additionally, normal in a medical context is generally defined as no deviation outside of the normal reference range for that age and sex, whilst diseases are seen as deviation from this normal condition for that age and sex (Boorse, 1975; Assaf et al., 2010). Thus, someone with a blood pressure of below 120/80 is seen as normal while a blood pressure above 140/90 or below 85/55 is abnormal and a sign of disease (WHO, 2015). The stratification of reference ranges for age is needed to distinguish fully developed adults from still developing children. This avoids, for example, the lack of sperm production in human male neonates being classified as a disease (Boorse, 1977). In contrast, stratifying elderly from younger aged adults is not based on any good biological argument but instead masks aging as separate from disease, despite it being apparent that aging represents a deviation of the more desired state of youthful physical and mental capacities (Callahan and Topinkova, 1998). Whilst a statement such as this could be considered ageist, such a conception is based on the misunderstanding of what is meant by youthful. Aging as the passage of time and the accumulation of wisdom is not undesirable; the physiological decline that accompanies the process, however, most certainly is (Mackey, 2003).

The vast majority of animal species undergo the process of aging. Whilst aging is a nearly universal occurrence, it should be noted that other medical problems such as muscle wastage leading to sarcopenia, reduction in bone mass and density leading to osteoporosis, increased arterial hardening resulting in hypertension, atherosclerosis, and brain tissue atrophy resulting in dementia, all of which are nearly universal in humans, are classified as diseases in need of medical interventions (Bierman, 1985; WHO, 1994; Izaks and Westendorp, 2003; Gems, 2011). Also, autopsy studies indicate that amyloidosis may be almost universal in elderly people (Blumenthal, 2002) and, in autopsies performed by the Supercentenarian Research Foundation (SRF), amyloidosis has been identified as the cause of death in about 70% of people over 110 years of age (Coles and Young, 2012). Should we remove amyloidosis from medical textbooks as an age-related disease just because it happens to occur in almost every elderly subject? It should be noted that some have indeed raised the question if senile amyloidosis should be seen as a disease (Blumenthal, 1995). David Gems notes that while aging is universal this fact does not exclude aging from being a disease but rather means that aging is a “special form of disease” (Gems, 2011). Even if one is to continue with the reasoning that aging is a natural process, this does not mean that because disease is seen as a transgression from a natural, normal state, aging should fall outside the scope of medicine. Other natural processes such as pregnancy, cosmetic issues and the like, although not defined as diseases, are accepted targets for medical intervention such as contraception, *in vitro* fertilization and cosmetic surgery (Boorse, 1975).

While most still seem to consider aging not to be a disease others have started to question this position. Some have argued that aging should be considered a disease (Caplan, 1992; Gems, 2003, 2011), a syndrome (Esser and Keller, 1976) or a “disease complex” (Perlman, 1953, 2003). Whilst many aging researchers have openly declared that the universality of the aging process means it is not a disease, aging fits the given medical definition of a disease. There is no disputing the fact that aging is a “harmful abnormality of bodily structure and function.” What is becoming increasingly clear is that aging also has specific causes, each of which can be reduced to a cellular and molecular level, and recognizable signs and symptoms (López-Otín et al., 2013).

The fluidity of what constitutes a disease and what is normal has recently been illustrated by the declaration by the American Medical Association House of Delegates that obesity is a disease (American Medical Association, 2013). Obesity, just like aging, does not comply with the traditional defining characteristics of disease. But yet to target an issue such as obesity, categorizing it as a disease makes the road to developing treatment a much easier one to take. As aging appropriately fits the definition of disease, there is a shifting consensus that aging should be seen as a disease process in itself, and not a benign progression of age that increases the risk of disease.

## The benefits of labeling aging as a disease

Callahan and Topinkova (1998) write: “In short, not only does aging lend itself to be characterized as a disease, but the advantage of doing so is that, by rejecting the seeming fatalism of the label “natural,” it better legitimizes medical efforts to either eliminate it or get rid of those undesirable conditions associated with it.” The goal of biomedical research is to allow people to be “as healthy as possible for as long as possible” (de Magalhães, 2014). Having aging recognized as a disease would stimulate grant-awarding bodies to increase funding for aging research and develop biomedical procedures to slow the aging process (Kelland, 2010). Indeed, Engelhardt states that calling something a disease involves the commitment to medical intervention (Engelhardt, 1975). Furthermore, having a condition recognized as a disease is important to have treatment refunded by health insurance providers (Reznek, 1987).

**Figure 1:**
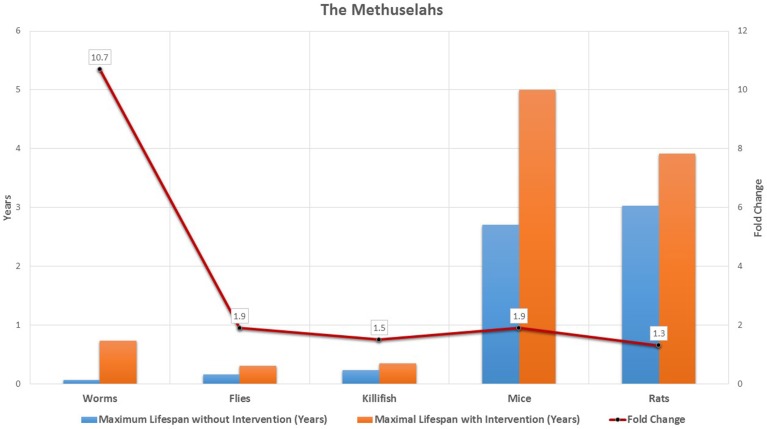
**The methuselahs in lab**. The increase in maximum lifespan in the laboratory was obtained in 5 animal species, both without any interventions, and by dietary, chemical, or genetic interventions. For each organism the impact of increase in maximum lifespan through intervention is indicated in the graph using fold change.

During the last 25 years, by targeting the underlying processes of aging biomedical scientists have been able to improve the health and lifespan of model organisms, from worms and flies, to rodents and fish. We can now consistently improve the lifespan of *C. elegans* by more than ten-fold (Ayyadevara et al., 2008), more than double the lifespan of flies and mice (Bartke et al., 2001; Sun et al., 2002), and improve the lifespan of rats and killifish by 30 and 59%, respectively (Valenzano et al., 2006; Zha et al., 2008) (see Figure 1 and Supplementary Table 1). Currently, our treatment options for the underlying processes of aging in humans are limited. However, with current progress in the development of geroprotective drugs, regenerative medicine, and precision medicine interventions, we will soon have the potential to slow down aging (Bulterijs, 2011, 2012). Finally, we should note that recognizing aging as a disease would shift anti-aging therapies from the Federal Drug Administration's (FDA) regulations for cosmetic medicine to the more rigorous regulations for disease treatment and prevention (Gems, 2011).

## Conclusion

We believe that aging should be seen as a disease, albeit as a disease that is a universal and multisystemic process. Our current healthcare system doesn't recognize the aging process as the underlying cause for the chronic diseases affecting the elderly. As such, the system is setup to be reactionary and therefore about 32% of total Medicare spending in the United States goes to the last 2 years of life of patients with chronic illnesses, without any significant improvement to their quality of life (Cooper, 1996; Neuberg, 2009). Our current healthcare system is untenable both from a financial and health and well-being prospective. Even minimal attenuation of the aging process by accelerating research on aging, and development of geroprotective drugs and regenerative medicines, can greatly improve the health and well-being of older individuals, and rescue our failing healthcare system.

### Conflict of interest statement

The Editor Zhavoronkov declares that, despite being affiliated to the same institution as the authors Raphaella S. Hull and Avi G. Roy, the review process was handled objectively and no conflict of interest exists. The authors declare that the research was conducted in the absence of any commercial or financial relationships that could be construed as a potential conflict of interest.
