# Supplementary material for: It is time to classify biological aging as a disease
Source: Front Genet. 2015 Jun 18;6:205. doi: 10.3389/fgene.2015.00205 (PMC4471741; doi:10.3389/fgene.2015.00205)
Supplement: Supplementary file 1 [file Table1.DOCX]

***Supplementary Material***

**It is time to classify biological aging as a disease**

Sven Bulterijs^1,2,*^, Raphaella S. Hull^3,5^, Victor C. E. Björk^2,4^, Avi G. Roy^2,5,6^

^1^ Faculty of Science, Ghent University, Ghent, Belgium.

^2^ Heales vzw, Brussels, Belgium.

^3^ Biochemistry Department, University of Oxford, Oxford, UK.

^4^ Institutionen för biologisk grundutbildning, Uppsala University, Uppsala, Sweden.

^5^ The Biogerontology Research Foundation, BGRF, London W1J 5NE, UK.

^6^ Institute for Translational Medicine, University of Buckingham , Buckingham , UK..

**Correspondence:**

Sven Bulterijs

Heales vzw

18 rue Jules Delhaize

Brussels, 1080, Belgium

[sven.bulterijs@heales.org](mailto:sven.bulterijs@heales.org)

| **Organism** | **Maximum Lifespan without Intervention (Years)** | **Maximal Lifespan with Intervention (Years)** | **Fold Change** | **Reference** |
| --- | --- | --- | --- | --- |
| **Worms** | 0.068 | 0.73 | 10.7 | Ayyadevara et al., 2008 |
| **Flies** | 0.16 | 0.3 | 1.9 | Sun et al., 2002 |
| **Killifish** | 0.23 | 0.35 | 1.5 | Valenzano et al., 2006 |
| **Mice** | 2.7 | 5 | 1.9 | Bartke et al., 2001 |
| **Rats** | 3.03 | 3.91 | 1.3 | Zha et al., 2008 |

**Supplementary Table 1.** **The methuselahs in lab**. Data used to construct Figure 1. The increase in maximum lifespan in the laboratory was obtained in 5 animal species, both without any interventions, and by dietary, chemical, or genetic interventions.
